# Pot economy and one-pot synthesis

**DOI:** 10.1039/c5sc02913a

**Published:** 2016-01-06

**Authors:** Yujiro Hayashi

**Affiliations:** a Department of Chemistry , Graduate School of Science , Tohoku University , 6-3 Aramaki-Aza Aoba, Aoba-ku , Sendai 980-8578 , Japan . Email: yhayashi@m.tohoku.ac.jp ; Fax: +81-22-795-6566 ; Tel: +81-22-795-3554

## Abstract

The one-pot synthesis of a target molecule in the same reaction vessel is widely considered to be an efficient approach in synthetic organic chemistry. In this review, the characteristics and limitations of various one-pot syntheses of biologically active molecules are explained, primarily involving organocatalytic methods as key tactics. Besides catalysis, the pot-economy concepts presented herein are also applicable to organometallic and organic reaction methods in general.

## Abstract 2

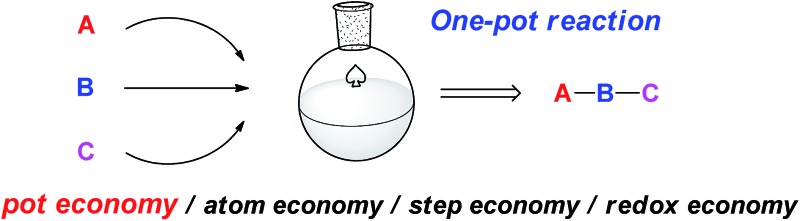
This review describes the importance and usefulness of pot-economy and one-pot reactions in current synthetic organic chemistry.

## 1. Introduction

Efficiency and environmental sustainability are central issues in contemporary organic chemistry. Both need to be addressed carefully when making a valuable target molecule over several distinct steps. When feasible, an effective approach is to synthesize the target in a single reaction vessel. This approach is often termed ‘one-pot’, and can apply to a multi-step reaction, method, or synthesis. It is effective because several synthetic transformations and bond-forming steps can be carried out in a single pot, while circumventing several purification procedures at the same time. A one-pot procedure can thus minimize chemical waste, save time, and simplify practical aspects. In fact, this approach has been used widely in synthetic organic chemistry for a long time. For instance, Robinson's one-pot synthesis of tropinone is a landmark achievement in organic chemistry, which was reported nearly 100 years ago (eqn (1)).^1^ Among many other classic examples involving single-pot procedures, one-pot reactions have been elegantly utilized in the biomimetic syntheses of progesterone by Johnson,^2^ endiandric acid by Nicolaou,^3^ and proto-daphniphylline by Heathcock.^4^

In the growing field of organocatalysis,^5^ organocatalysts are particularly effective reagents in achieving a one-pot, multi-step synthesis. This is evidenced by a dramatic increase of impressive syntheses over the past decade.^6^ For example, Enders reported a breakthrough one-pot synthesis of a chiral cyclohexenecarbaldehyde with excellent enantioselectivity using a diphenylprolinol silyl ether-mediated^7,8^ Michael reaction as a key step (eqn (2)).^9^ This catalyst was independently developed by our group^7^ and the group of Jørgensen.^8^ Herein, we review the characteristics of one-pot syntheses employing prolinol-based organocatalysts as developed in our laboratories. By detailing the criteria for a successful multi-step synthesis, the insights and concepts presented are intended to be transferable to other organic reaction methods, synthetic strategies, and the field of targeted synthesis in general.1
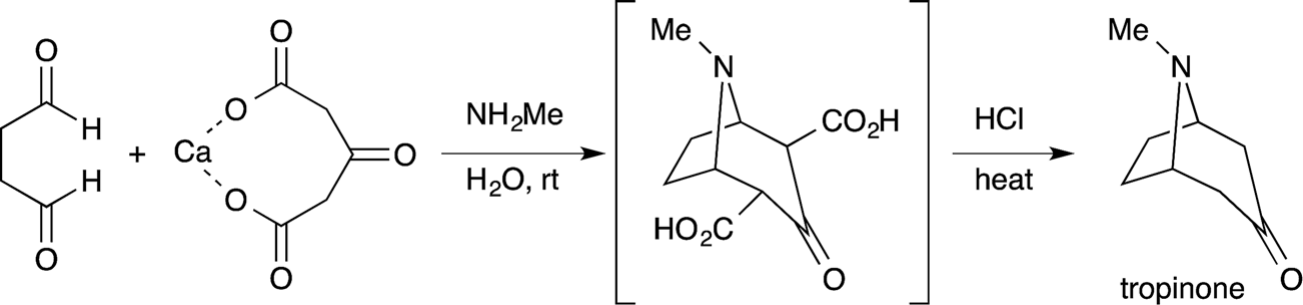

2
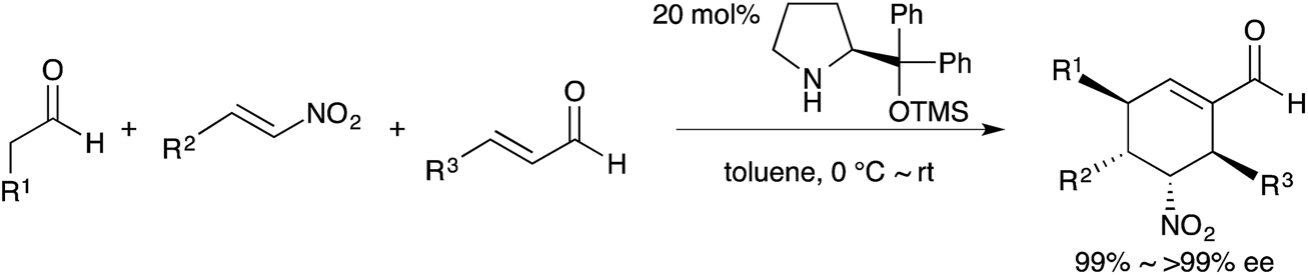



## 2. One-pot/domino/cascade/tandem reaction

There are several terminologies to describe multi-step reactions that take place in one pot. These include: “domino reaction”, “cascade reaction”, and “tandem reaction”. Nicolaou pointed out that these descriptions are comparatively interchangeable in his 2006 review titled “Cascade Reactions in Total Synthesis”.^10^ Tietze suggested the usage of “domino reaction” rather than “cascade reaction” or “tandem reaction”, and defined a domino reaction as a process involving two or more bond-forming transformations (usually C–C bonds) that take place under the same reaction conditions without adding additional reagents and catalysts, and in which subsequent reactions result as a consequence of the functionality formed in the previous step.^11^ Denmark proposed to keep the all-encompassing definition of “tandem reactions” as reactions that occur one after the other, and to use the modifiers cascade (or domino), consecutive, and sequential to specify how the two (or more) reactions follow.^12^ Fogg classified one-pot processes as one-pot reaction, domino catalysis, or tandem catalysis, the latter of which being further subdivided into orthogonal catalysis, auto-tandem catalysis, and assisted-tandem catalysis.^13^

In spite of all these terminologies, a one-pot synthesis is defined as a strategy to improve the efficiency of a chemical reaction, whereby a reactant is subjected to successive chemical reactions in just one reactor.^14^ As long as a particular sequence of reactions is carried out in the same reactor, it is considered to be “one-pot” in this review. Thus, a one-pot synthesis has a much wider meaning than a cascade, domino or tandem reaction, and the concept of a one-pot synthesis encompasses all such reaction types as well as the multi-step strategies that are adopted in a single vessel or reactor.

## 3. Pot economy/atom economy/step economy/redox economy

‘Green’^15^ and ‘efficiency’ are two principal issues in science and industry. These issues can be characterized in terms of atom economy, step economy, and redox economy.^16^ Atom economy was proposed by Trost, who stated that synthetic methods should be designed to maximize the incorporation of all materials used in the process into the final product (ACS Green Chemistry Principle #2).^17^ Reactions with no byproducts^18^ are thus desirable and it is necessary to employ “clean” and reliable reactions when planning the synthesis of a target molecule. Step economy was proposed by Wender and is another fundamental economy to consider in order to minimize the number of reaction steps to a target molecule; this thereby reduces the length, cost, development time, execution time, effort, separation methods, and environmental impact of a synthesis.^19^ Step economy is clearly influenced by selecting the right reaction method and sequence to allow for an optimal increase in target-relevant complexity. Redox economy was recently proposed by Baran and Hoffmann and relates to minimizing unnecessary changes in the oxidation states of isolatable intermediates, and thereby relates to reducing the number of steps in a given synthetic sequence.^20^ The concepts of redox and step economy are thus important in the synthetic design and strategic implementation of reaction methods to a target molecule.

An additional economy to consider is in the number of pots required for each reaction method. In other words, the workup and isolation of intermediates is not always necessary. Indeed, chemists have omitted workup procedures and carried out several reactions in the same reactor for a long time. When several reactions are conducted in a single reactor, without isolating or purifying the intermediates, we can reduce the amounts of solvent, waste, time, labour and cost. Thus, “pot-economy”^21^ is also important in synthesizing a target molecule in terms of ‘greenness’ and practicability.

Thus, in realizing a multistep synthesis, chemists first retro-synthesize the target molecule, in which they choose a synthetic strategy and reaction sequence according to the principles of step and redox economy. Next, appropriate reagents and reaction conditions are selected according to atom economy. Today, during these design and development stages, chemists are now adopting the principles of pot economy. Such aspects are discussed next.

## 4. Telescope reaction/one-pot reaction

Process chemists often carry out several reactions in a single reactor without the need to isolate intermediates. This multi-step approach is termed as a “telescoped reaction”. According to Dr J. Dunetz at Gilead Sciences,^22^ a telescoped reaction is defined as follows: while there is no formal definition, in general terms, telescoping is the execution of multiple transformations (including quenches and other workup operations) without the direct isolation of intermediates. Telescoped solutions of intermediates can be extracted, filtered (as long as the product remains in the filtrate), and solvent exchanged, but the intermediate is ultimately held in solution and carried forward to the subsequent transformation. These workup operations add to the expense of a process, and the best telescoped processes will minimize solvent exchange, *etc.*

As long as a reaction sequence is conducted in the same reactor, this approach is a one-pot reaction. However, telescoped reactions and one-pot reactions are not always the same. For instance, the (–)-oseltamivir synthesis given in Section 8.1 (Scheme 2),^23^ is regarded as a one-pot reaction but this synthesis is not telescoped due to the need to concentrate reactor contents to dryness in order to replace solvents. Such work-up steps are regarded as an isolation of the crude intermediates or product material. The one-pot synthesis of (–)-oseltamivir given in Section 8.2 (Scheme 3),^24^ however, is regarded as both one-pot and telescoped, because all reagents are added successively to one reactor without the need to evaporate or replace solvents.

## 5. Reaction/step, intermediate, and byproduct/side-product

Before discussing one-pot reactions, it is necessary to clarify the usage of several terminologies such as “reaction”, “step”, “intermediate”, “byproduct” and “side-product”.

In this review, we follow the definition of “reaction step” according to the IUPAC gold book,^25^ namely: an elementary reaction, constituting one of the stages of a stepwise reaction in which a reaction intermediate (or, for the first step, the reactants) is converted into the next reaction intermediate (or, for the last step, the products) in a sequence of intermediates between reactants and products.

“Intermediate” is defined according to the same book^25^ as a molecular entity with a lifetime appreciably longer than a molecular vibration (corresponding to a local potential energy minimum of a depth greater than RT) that is formed (directly or indirectly) from the reactants and reacts further to give (either directly or indirectly) the products of a chemical reaction. Thus, it is a non-transient, isolatable reaction product.

According to these definitions, reactions and reaction steps are not always the same: for instance, the aldol condensation reaction consists of two reaction steps, because there are two elementary reactions involved, namely, an aldol reaction and a dehydration reaction.

As for the terms “byproduct” and “side-product”, we follow the definitions of Watson,^26^ namely: byproducts are materials that are produced as a direct result of the desired reaction, and so they will appear as part of the fully balanced chemical equation. Side-products, on the other hand, are the result of side reactions.

## 6. Effective reactions for a one-pot synthesis

There are certain reaction criteria that are effective for a one-pot synthesis of a target molecule. These include reactions, in which: (1) the intermediate compound is unstable; (2) the intermediate compound is malodorous, hazardous, or toxic; (3) there is equilibration of intermediate compounds; (4) there is equilibration of the starting material and the intermediate compound; (5) the side-products generated are convertible into the desired intermediate compound or final product; (6) the same reagents are employed in subsequent reactions. Each reaction criteria will be explained in this section.

### 6.1 Reaction in which the intermediate compound is unstable

After the reaction to prepare compound C *via* an unstable intermediate B, the isolation of the unstable compound B would reduce the yield (eqn (3)). It would thus be better to carry out reaction 2 successively in the same reactor. In this review, when the intermediate compound B is used directly in the next reaction, in the same pot without isolation, we characterise this intermediate as [B], whereby the compound is given in square brackets (eqn (3)).3
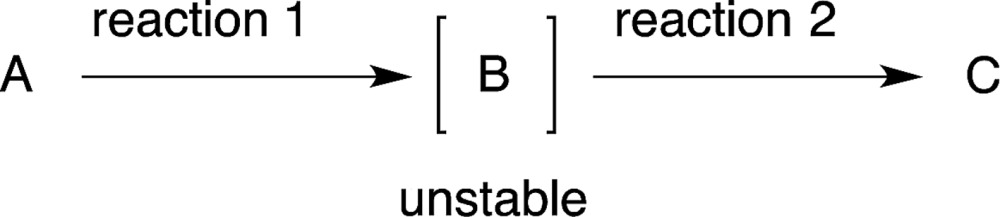



An example of this case is found in our second generation-synthesis of (–)-oseltamivir (eqn (4)).^22^ The Michael reaction of aldehyde **2** and nitroalkene **3** afforded the Michael product **4** with excellent diastereo- and enantioselectivity. If isolated, this intermediate **4** was readily isomerized which reduced the resultant *syn*/*anti* selectivity for product **6**. The next reaction is a domino Michael/Horner–Wadsworth–Emmons reaction. If the Michael reaction was subjected to work-up, the yield of **6** would be significantly reduced, due to the isomerization of the desired *syn*-intermediate to the undesired *anti*-isomer. Thus, a one-pot reaction sequence was essential for an optimal yield of product **6**.4
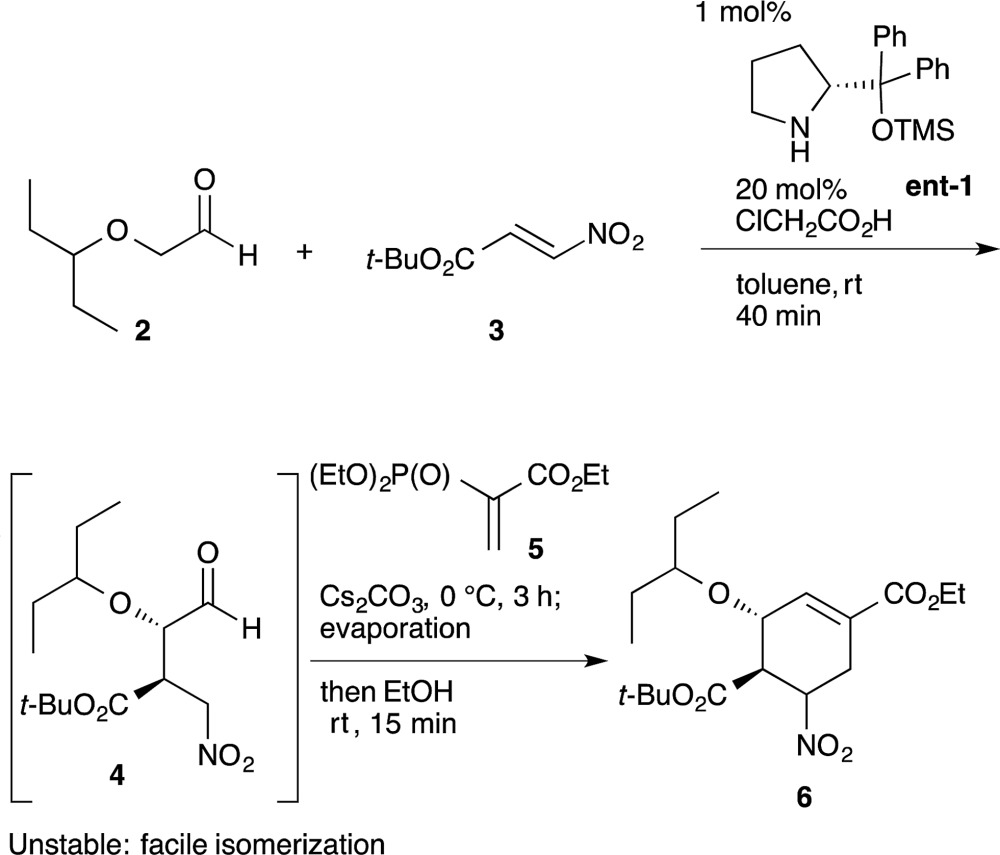



### 6.2 Reaction in which the intermediate compound is malodorous, hazardous, or toxic

It is a synthetic advantage to carry out experiments without the need to remove or isolate intermediates with bad smells or that present high safety risks (*e.g.*, explosion hazard, toxicity). In our second generation synthesis of (–)-oseltamivir,^27^ as given in Section 8.1 (Scheme 2), an acyl azide intermediate was identified as a potential hazard. We thus carried out the preparation of the azide intermediate and subsequent rearrangement reaction without workup, giving synthetic merit to the one-pot procedure.

### 6.3 Reaction in which there is equilibration between intermediate compounds


5
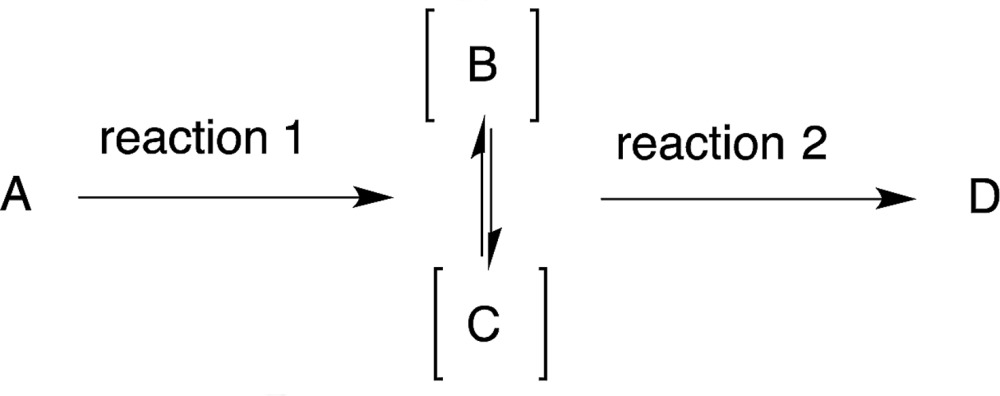
For reactions A → D that produce interchangeable intermediates (B or C) that are both capable of producing the desired product D, there is no need to isolate or separate the intermediates (eqn (5)). In our one-pot synthesis of chiral bicyclo[3.3.0]octatriene (eqn (6)), the diphenylprolinol silyl ether-mediated Michael reaction of cinnamaldehyde (**7**) and cyclopentadiene (**8**) was first performed to afford the Michael product **10** with 70 : 30 diastereoselectivity.^28^ Addition of a base promotes the intramolecular nucleophilic addition of cyclopentadienyl anion **11** to the formyl moiety, followed by dehydration, to afford the bicyclo[3.3.0]octatriene **12** in good yield with excellent enantioselectivity. In this reaction sequence, it is not necessary to separate the diastereomers in the first reaction because the same cyclopentadienyl anion **11** can be generated from either Michael product **10a** or **10b**.6
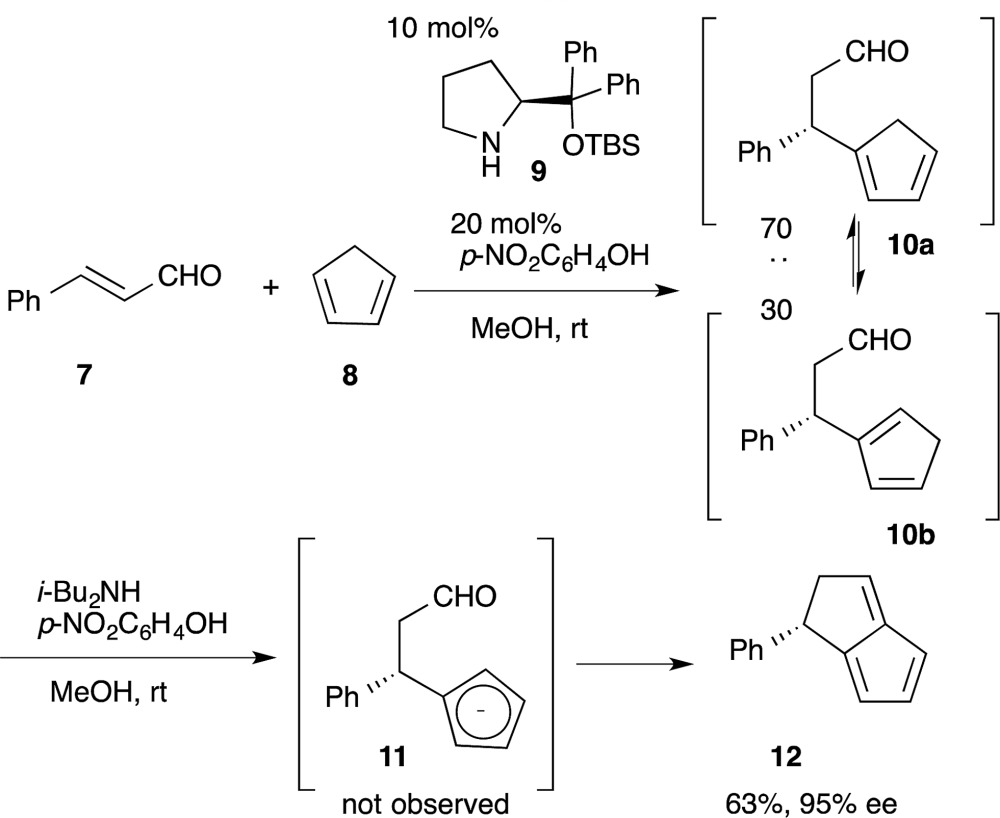



Another example is illustrated in the synthesis of (–)-oseltamivir (eqn (7)). Here, the domino Michael/Horner–Wadsworth–Emmons reaction starting from nitroaldehyde **4** and ethyl acrylate **5** proceeded to afford the ethyl cyclohexene carboxylate derivative **6**, of which a mixture of 5(*R*)- and 5(*S*)-isomers was obtained. After the subsequent addition of toluenethiol, Michael addition proceeded with epimerization of C-5 to provide the 5(*S*)-isomer **13** selectively. As both **6a** and **6b** afforded the same compound **13**, it is not necessary to separate the diastereomers **6a** and **6b**. It is thus a synthetic advantage to carry out such successive reactions in the same rector.7
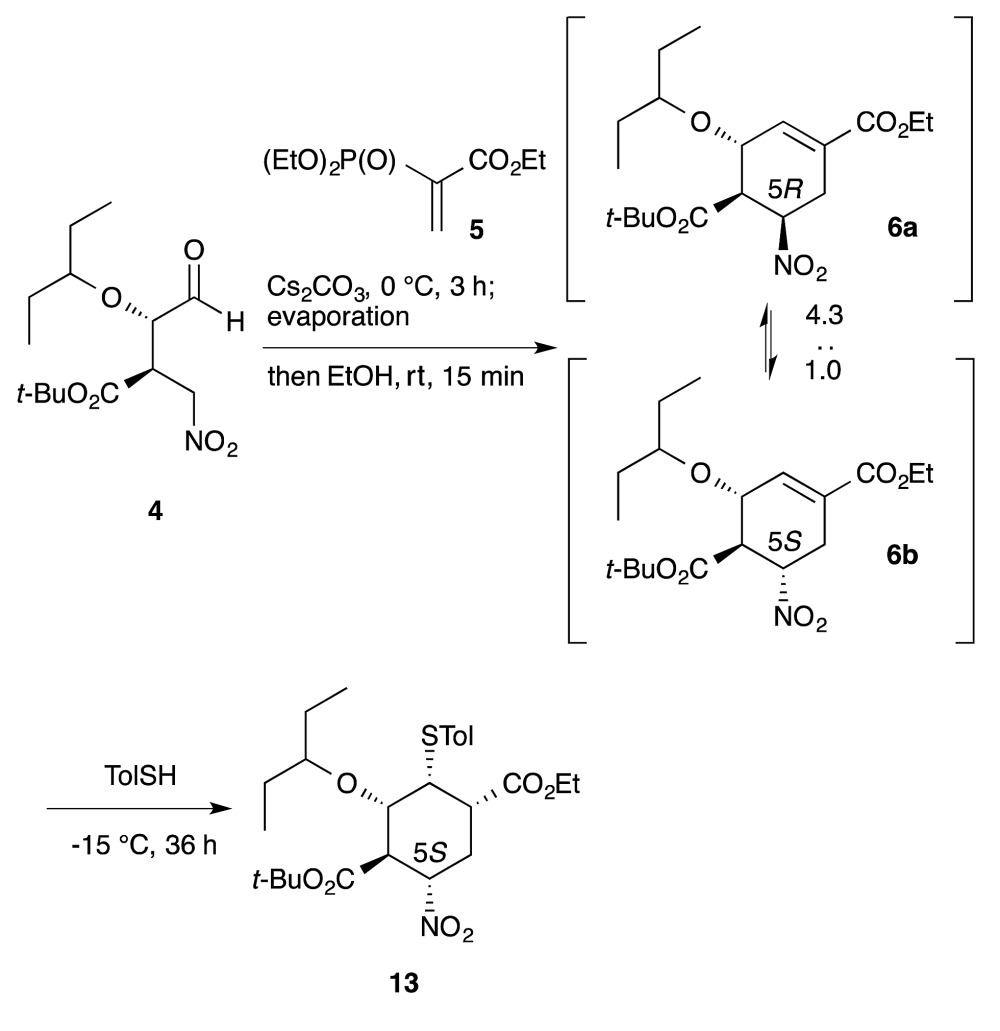



### 6.4 Reaction in which there is equilibration of the starting material and the intermediate compound


8
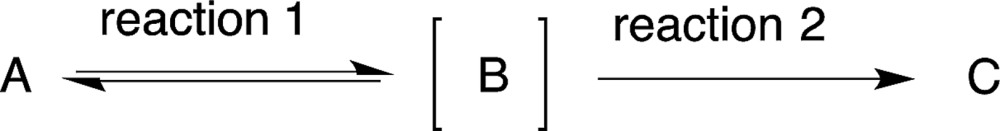
A one-pot, two-reaction procedure to produce a product C is also advisable where the intermediate [B] from reactant A can reverse back into A over time (eqn (8)). Rovis reported the reaction of crotonaldehyde (**14**) and acetylacetone (**15**) *via* a Michael addition reaction followed by an intramolecular cross-benzoin condensation (eqn (9)). Compared to the orthogonal use of two organocatalysts in a single reactor, the yield and enantioselectivity decreased when these reactions were conducted in a two-pot isolated sequence.^29^ Thus, the second catalyst served to drive the intermediate **17** to the product **19** by diminishing the possibility of a retro-Michael reaction occurring.9
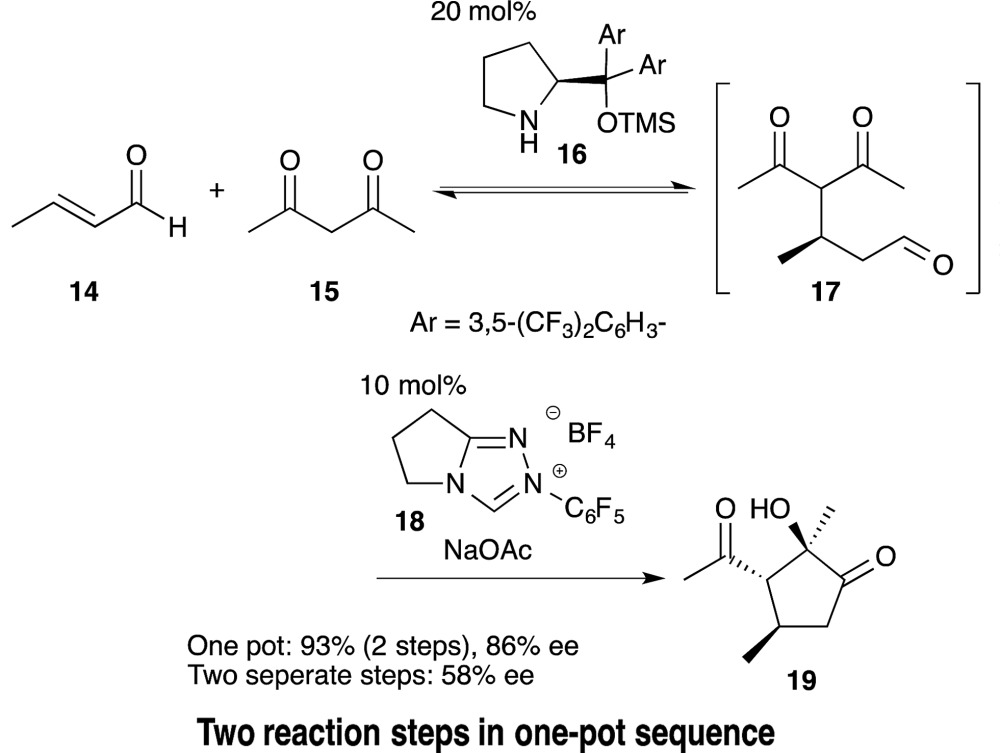



### 6.5 Reaction in which the side-products generated are convertible into the desired intermediate compound or final product

In reactions where a side-product C is generated from the reactant A by reaction 1, but C is also convertible to the desired product B *via* reaction 2 within the reaction mixture that is generated, both reactions 1 and 2 can be carried out successively in the same reactor without the need to separate compounds B and C (eqn (10)).10
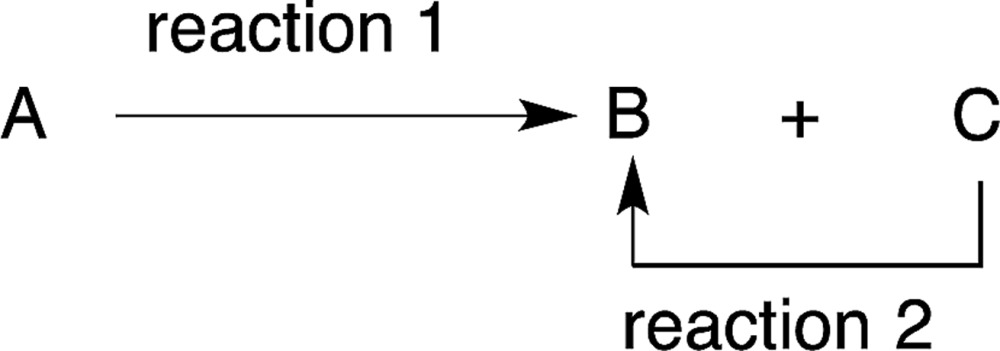



In our synthesis of (–)-oseltamivir,^23,24,27^ when the nitro compound **4** was treated with **5** in CH_2_Cl_2_ with Cs_2_CO_3_, a domino Michael/Horner–Wadsworth–Emmons reaction proceeded to afford the desired product **6**, as well as the undesired side-products **20** and **21** (eqn (11)). Compound **20** is a doubly-reacted Michael product formed by the further reaction of **6** with **5**, and **21** possesses a *trans*-stereochemistry about the hydroxyl group and phosphoric ester moiety, from which *syn*-elimination does not proceed. As it was found that both side-products **20** and **21** can be converted into the desired product **6** in EtOH with Cs_2_CO_3_ (*via* a retro-Michael reaction from **20** or a retro-aldol/Horner–Wadsworth–Emmons reaction from **21**), the product **6** could be obtained in a yield approaching 80% in an efficient manner by conducting both reactions 1 and 2 in the same reactor successively.11
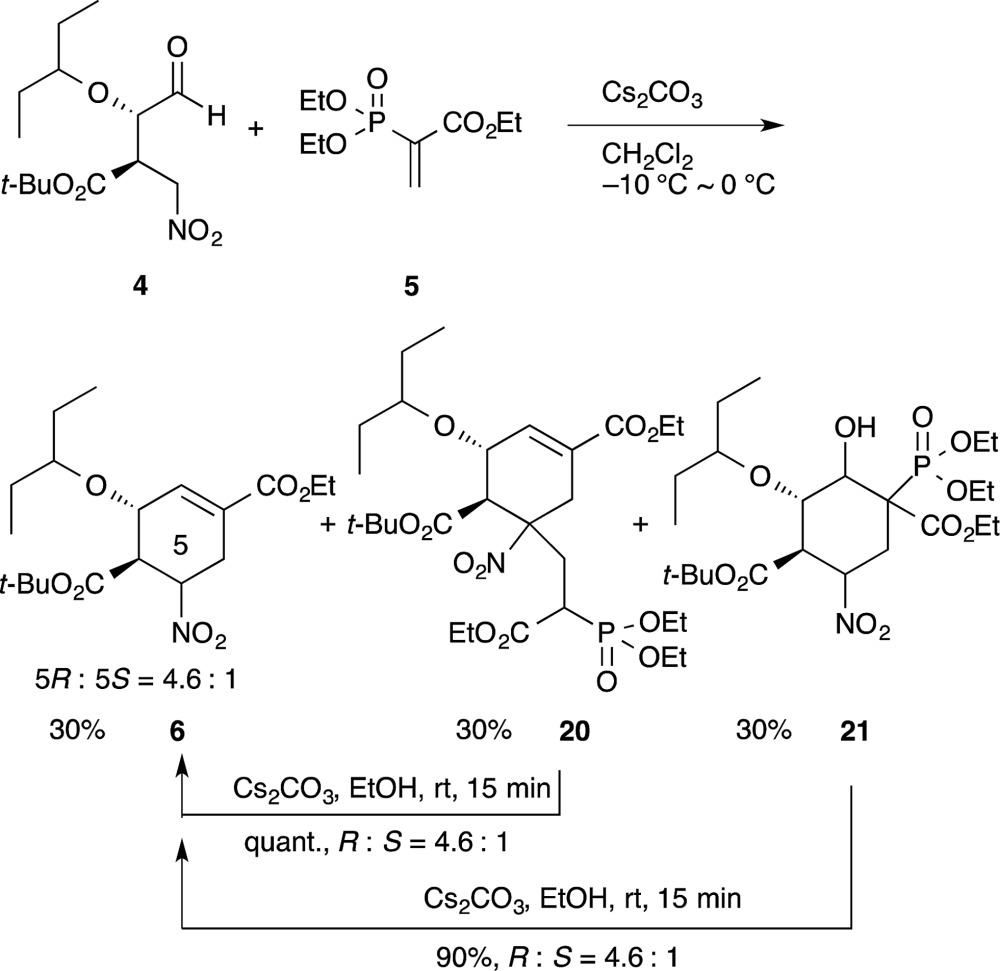



### 6.6 Reaction in which the same reagents are employed in subsequent reactions


**Scheme 1:**
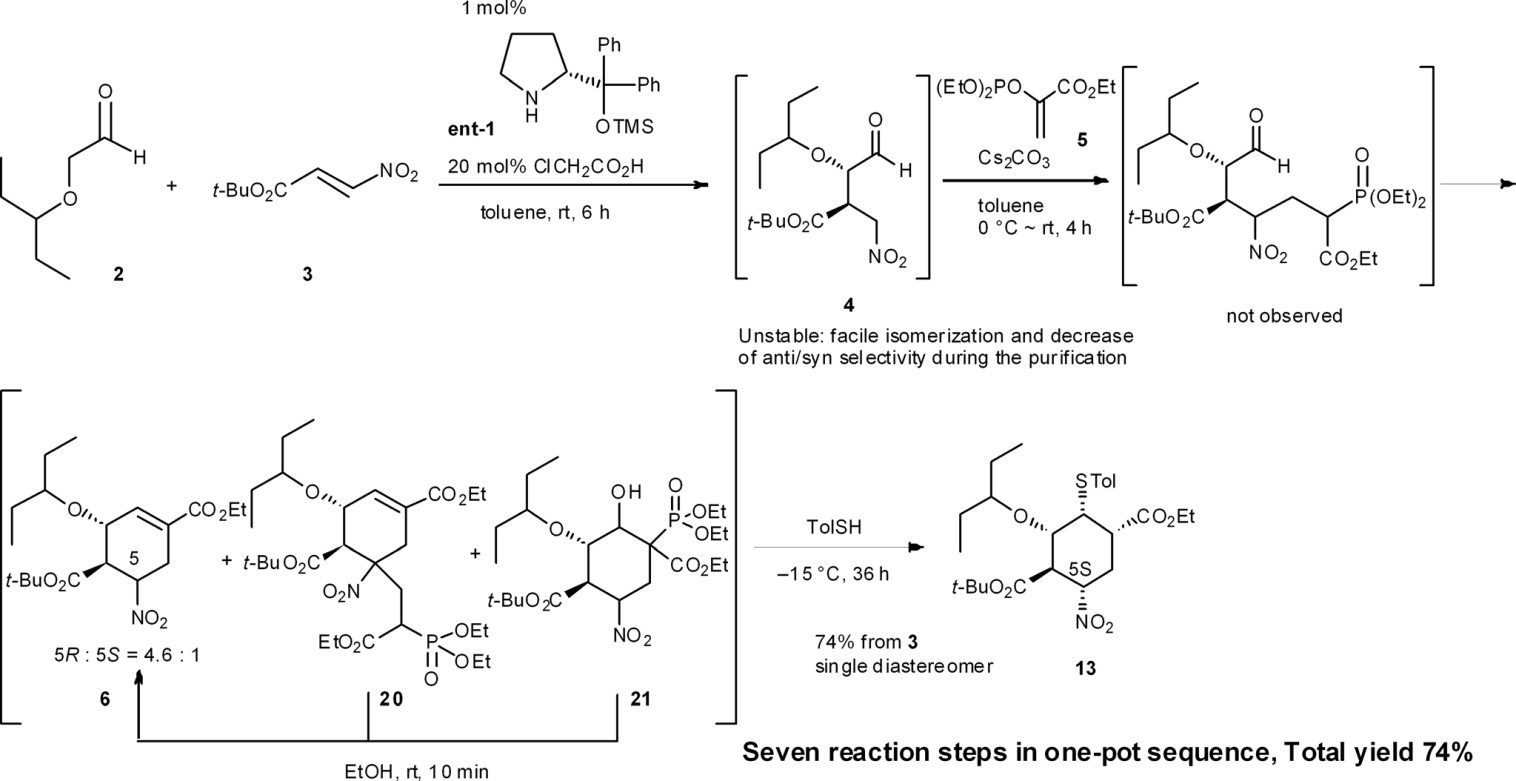
One-pot synthesis of **13** during the synthesis of (–)-oseltamivir.

12
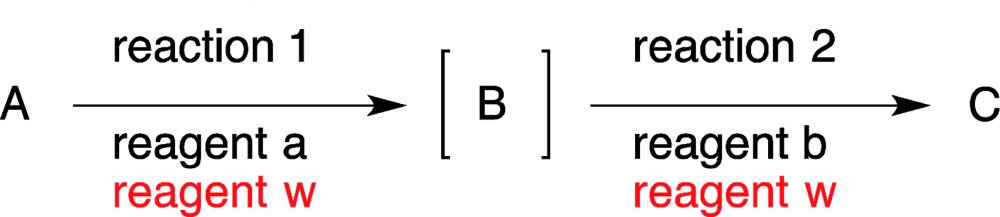
If the same reagent is employed in successive reactions, it is a synthetic advantage to carry out the two reactions in the same reactor (eqn (12)). An example is shown in our first one-pot reaction sequence to make (–)-oseltamivir, whereby Cs_2_CO_3_ was utilised in five different ways as a base (*vide infra*, Section 8.1, Scheme 1). Another example is found in the syntheses of (–)-horsfiline and (–)-coerulescine, where Zn acted in several ways (*vide infra*, Section 8.6).

## 7. Restrictions for one-pot reactions

There are several restrictions to carrying out a one-pot synthesis. These are detailed next.

### 7.1 Reaction

In a one-pot sequential synthesis of a target product, each reaction has to proceed in excellent yield, in which the generation of byproducts and side-products is minimized as much as possible. As the number of reactions increases, byproducts and side-products accumulate more and more affecting the following reactions and diminishing yields. Subsequent reactions thus have to proceed in the presence of these accumulated byproducts and side-products.

It is therefore important to select reaction methods with the least amount of byproducts and side-products. For instance, the Horner–Wadsworth–Emmons reaction is widely used in the synthesis of alkenes, but it produces an equimolar amount of phosphoric acid derivatives and such byproducts can interfere with successive reaction conditions due to acidity issues. Thus, it is important to not only select suitable reactions with minimal byproducts, but also to optimize reaction methods so as to suppress the generation of undesired side-products.

### 7.2 Solvent

If a solvent with a high boiling point is employed in a previous reaction, this may be unsuitable or suboptimal for the next reaction. Besides the solvent being difficult to remove completely *in vacuo*, the higher evaporation temperature may harm the structural integrity of the product *via* thermal decomposition. It is thus necessary to determine reaction conditions under which both reactions can proceed in the same solvent, or to employ a solvent with a low boiling point that can then be readily removed *via* evaporation.

### 7.3 Amount of reagent

When excess reagents are first employed, which cannot be removed under reduced pressure, the next reaction has to be carried out in the presence of this reagent and the following reaction conditions need to be compatible. It is thus desirable to use reagents with a low boiling point and in stoichiometric amounts relative to the reactant. Alternatively, the remaining reagents can be deactivated before the next reaction conditions are applied.

Reactions that give few byproducts and side-products are thus suitable for one-pot synthetic sequences, especially those that proceed with stoichiometric reagents and future-compatible low boiling solvents. Although certain reactions might afford undesired side-products, or reagents might remain in the reaction mixture, we can control the reactivity of these side-products or the remaining reagents by the addition of additives to make the next reaction or reactions work successfully. Thus, a “one-pot” reaction is not a simple combination of each, separately optimized, set of reaction conditions, but an additive sequence of reagents, solvent-modifications, and *in situ* quenching events.

Traditional “stop-and-go” synthesis is composed of a reaction, workup, and purification. Compared with this traditional approach, a one-pot synthesis is relatively difficult to optimize. Similarly, when compared to a sequence of solid phase reactions, in which excess reagents and high boiling solvents from the prior steps can be readily removed by washing with the next solvent, a one-pot reaction sequence is challenging to develop in practice.

## 8. Case studies: representative examples of one-pot syntheses

### 8.1 Two-pot synthesis of (–)-oseltamivir^23^

(–)-Oseltamivir phosphate (Tamiflu®), a neuraminidase inhibitor, is one of the most effective drugs for the treatment of influenza.^30^ Our group has a continuing interest in supplying this drug in an efficient manner, and have reported three versions of its synthesis. In the first and second generation approaches in 2009 and 2010, the highly substituted cyclohexane carboxylate **13** was synthesized with an initial set of sequential reactions in one pot (Scheme 1).^23,27a^

Here, the first reaction is the asymmetric Michael addition reaction between α-alkoxyaldehyde **2** and nitroalkene **3** catalyzed by diphenylprolinol silyl ether. Next, the Michael addition of the anion of nitroalkane **4** to the ethyl acrylate derivative **5** provided three compounds **6**, **20** and **21**. As the undesired side-products **20** and **21** can be transformed into the desired ethyl cyclohexenyl carboxylate **6** by adding EtOH solvent, **6** can be derived from both **20** and **21** (*cf.* the synthesis of **6** from **2** and **3** in Sections 6.1 and 6.5). The subsequent transformation of **6**, involving the stereoselective isomerization of the 5*R*-isomer **6a** and 5*S*-isomer **6b** in the same reactor, completed the synthesis of the highly substituted cyclohexane derivative **13** (as explained in Section 6.3).

In this one-pot reaction sequence to **13**, a highly functionalized chiral cyclohexane framework with the correct relative and absolute configuration was synthesized over seven reaction steps: (1) a diphenylprolinol silyl ether-mediated, asymmetric Michael reaction (**2** and **3** → **4**), (2) a domino Michael reaction and (3) Horner–Wadsworth–Emmons reaction (**4** → **6**) combined with (4) a retro-aldol/(3) Horner–Wadsworth–Emmons elimination (**21** → **6**), (5) a retro-Michael reaction (**20** → **6**), (6) a base-catalyzed isomerization, and (7) a thiol-Michael reaction (**6** → **13**).

It should be noted that the same reagent was employed in several reactions in a single reactor, whereby changes in the solvent and temperature were used to moderate the reactivity of the intermediate compounds (Section 6.6). For instance, Cs_2_CO_3_ acts as a base in five different ways: (1) for the Michael reaction of nitroalkane **4** and vinylphosphonate **5** in toluene; (2) for the intramolecular Horner–Wadsworth–Emmons reaction; (3) in EtOH, for the retro-Michael reaction from **20** to **6** and retro-aldol/Horner–Wadsworth–Emmons-based reactions from **21** to **6**; (4) for the C5 isomerization of **6** for (5) the Michael reaction of the thiol and **6**, in which the reaction was conducted at a lower temperature (–15 °C) to suppress over-reaction, such as the elimination of HNO_2_ from **6**. It is thus operationally simple and economically advantageous to use the same reagent multiple times in the same reactor.

**Scheme 2:**
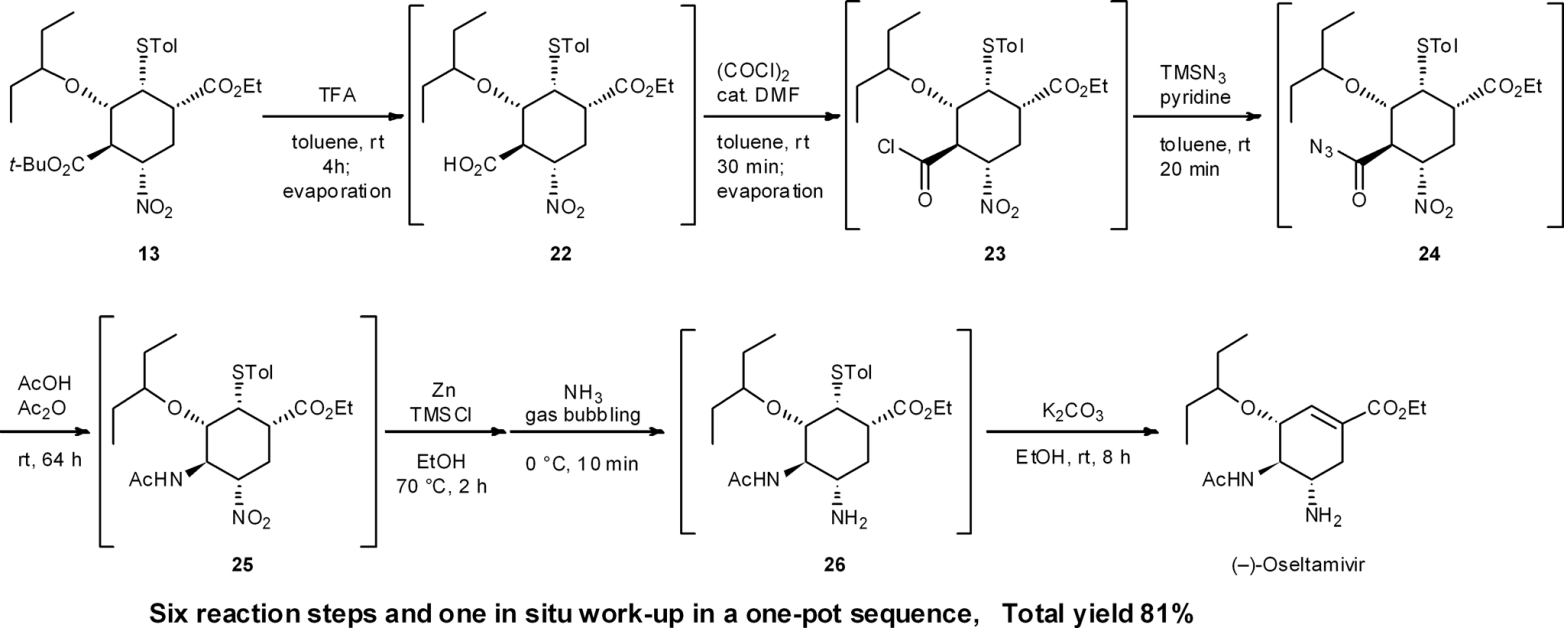
One-pot synthesis of (–)-oseltamivir from **13**.

During our second generation synthesis of (–)-oseltamivir, six reaction steps were conducted in a second, one-pot reaction sequence from **13** (Scheme 2). These include: (1) deprotection of a *tert*-butyl ester (**13** → **22**) and (2) its conversion to the acid chloride **23**, then (3) transformation to the acid azide **24**, (4) the domino Curtius rearrangement/amide formation of **25**, (5) the nitro reduction of **25** to the amine **26**, and (6) the retro-Michael reaction of the thiol group. In this case, several evaporation procedures were employed to change solvents and remove volatile components from the reaction mixture. After the reduction of the nitro moiety with Zn, NH_3_ bubbling is necessary to cleave zinc chelates *in situ*. This is comparable to a “stop-and-go” synthesis, whereby the Zn chelates are cleaved by acid treatment during aqueous workup. In the one-pot sequence, this kind of “*in situ* work-up” modification is necessary to carry out sequential one-pot reactions successfully. It is also worth mentioning that there is no need to isolate the potentially hazardous azide intermediate **24**, which is an advantage safety-wise (Section 6.2).

### 8.2 One-pot synthesis of (–)-oseltamivir^24^

**Scheme 3:**
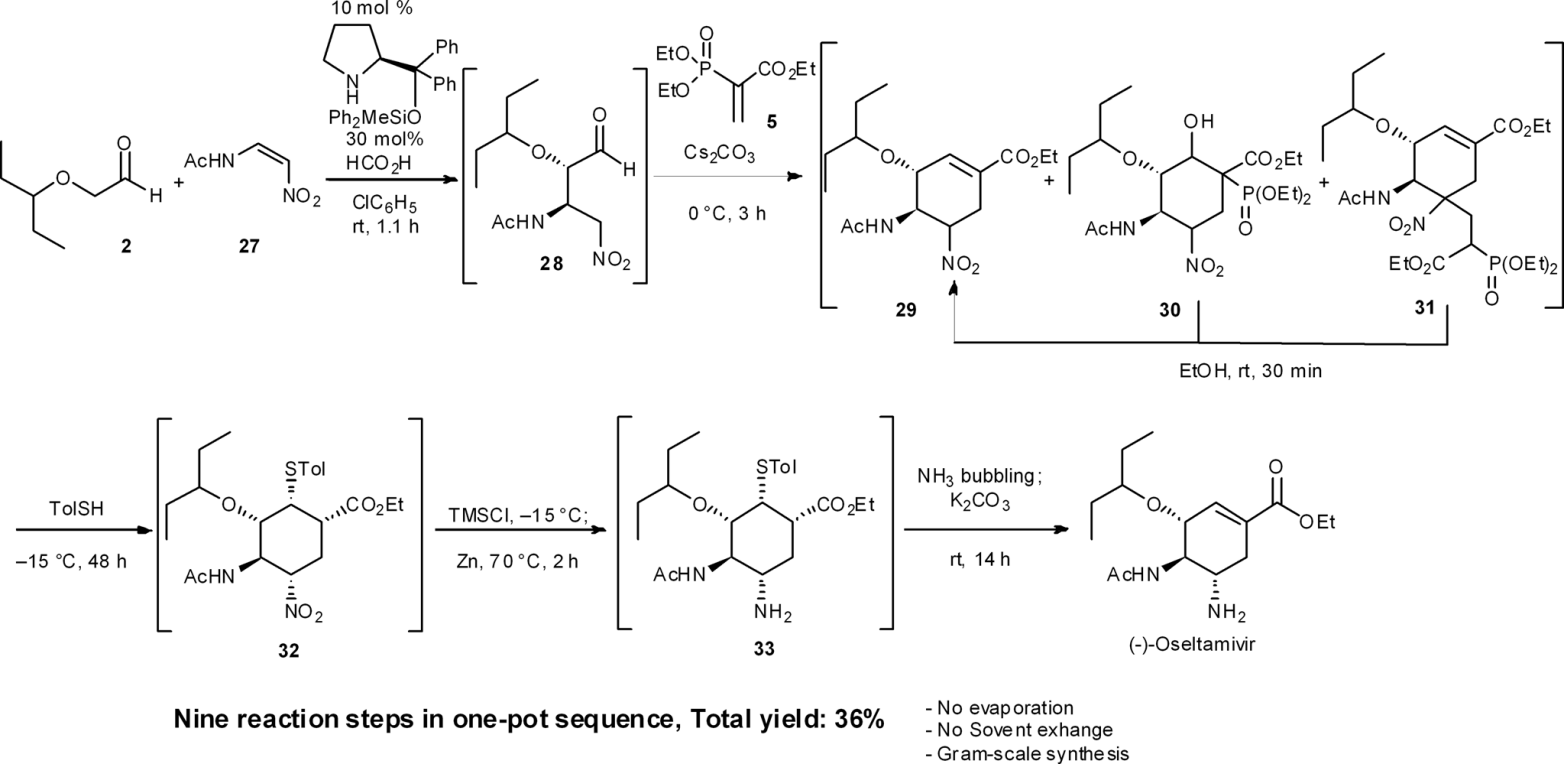
One-pot targeted synthesis of (–)-oseltamivir.

Recently, we accomplished a one-pot targeted synthesis of (–)-oseltamivir (Scheme 3),^24^ which is based on our own previous work and the syntheses of Ma,^31^ Sebesta^32^ and Lu.^33^ Starting from the diphenylprolinol silyl ether-mediated asymmetric Michael reaction of α-alkoxyaldehyde **2** and (*Z*)-*N*-2-nitroethenylacetamide **27**, the rest of the transformations are slight modifications of our previous synthetic sequences. Today, we can thus synthesize (–)-oseltamivir in a one-pot sequence in 36% yield by changing conditions only 6 times over nine reaction steps, including: (1) a diphenylprolinol silyl ether-mediated, asymmetric Michael reaction (**2** and **27** → **28**), (2) a domino Michael reaction/(3) Horner–Wadsworth–Emmons reaction (**28** → **29**), combined with (4) a retro-aldol/(3) Horner–Wadsworth–Emmons elimination (**30** → **29**), (5) a retro-Michael reaction (**31** → **29**), (6) a base-catalyzed isomerization, (7) a thiol-Michael reaction (**29** → **32**), (8) reduction of the nitro group to an amine (**32** → **33**), and finally (9) a retro-Michael reaction of the thiol.

A noteworthy synthetic advantage is that this one-pot synthesis does not involve any evaporation processes or solvent swapping. Also, the present synthesis is the first example of a stereochemically complex drug being synthesized in a single reactor, in significant yield, without the need to evaporate or swap solvents. This achievement shows the power of a carefully developed one-pot reaction sequence.

### 8.3 One-pot synthesis of ABT-341 ^34^

**Scheme 4:**
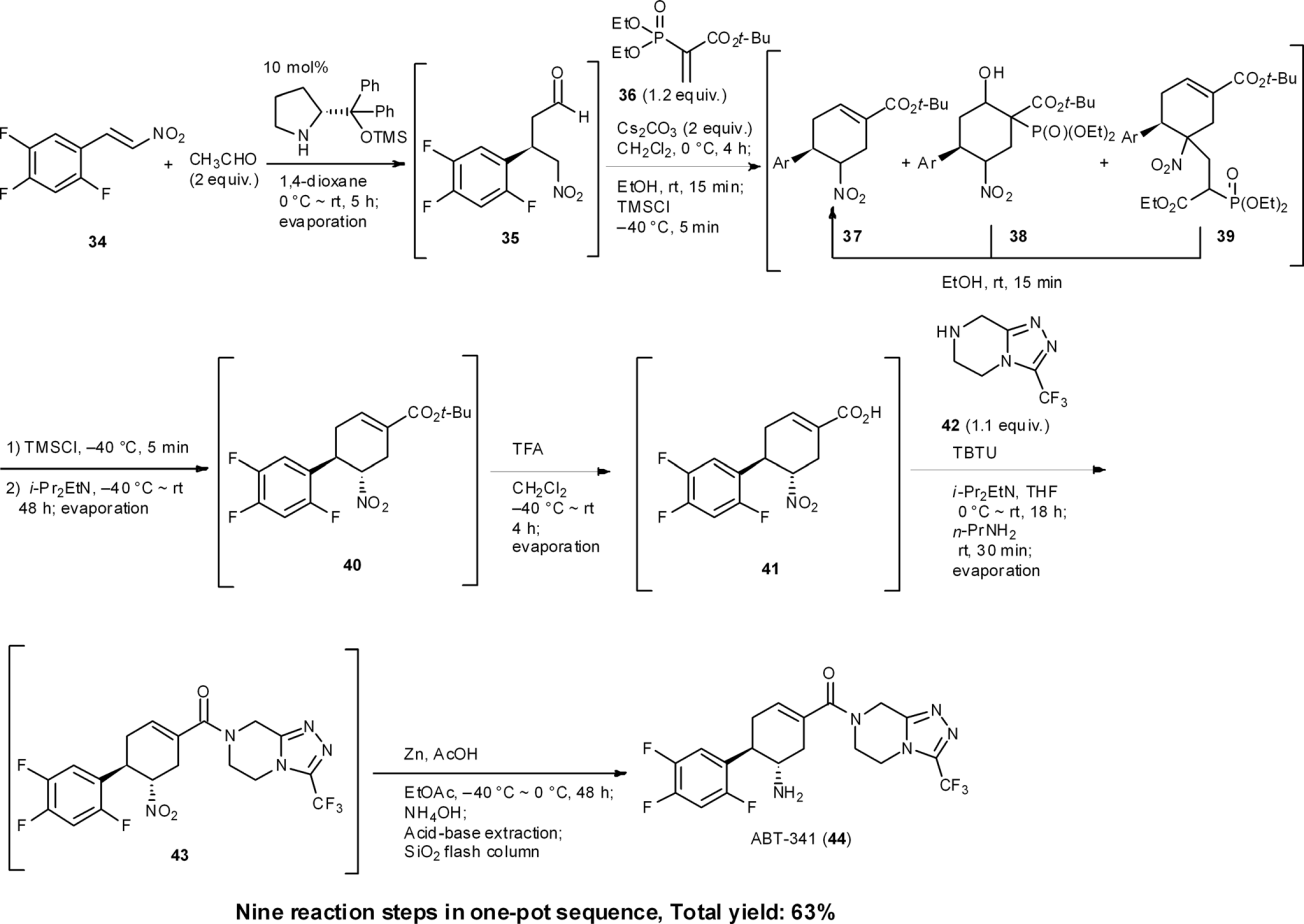
One-pot synthesis of ABT-341.

ABT-341 is a highly potent, selective, and orally bioavailable DPP4 (dipeptidyl peptidase IV) inhibitor, which is a drug candidate for type 2 diabetes being developed by Abbott Laboratories.^35^ We accomplished a one-pot sequential synthesis of ATB-341 based on an organocatalyst-mediated Michael reaction as a key step (Scheme 4). The first reaction is the diphenylprolinol silyl ether mediated asymmetric Michael reaction of acetaldehyde and nitroalkene **34** previously developed by our group.^36^ The successive reaction for the formation of chiral cyclohexene carboxylate **37** involves a method that we developed for the synthesis of (–)-oseltamivir, after which the isomerization of the α-position of the nitro group presented difficulties. Optimization of this isomerization with isolated **37** revealed that *i*-Pr_2_EtN cleanly afforded **40**. In a one-pot sequential reaction, however, no isomerization occurred. The differences between the one-pot and “stop-and-go” reaction sequences were reasoned to be due to the presence of Cs_2_CO_3_ preventing a reversible isomerization of the α-position of the nitro group. As Cs_2_CO_3_ cannot be removed without a standard work-up, as is the case for stop and go reactions, we investigated its deactivation (an *in situ* quenching step). In this event, we found that the addition of EtOH and TMSCl was key to deactivating Cs_2_CO_3_. That is, TMSCl reacts with the solvent, EtOH, to generate HCl, which reacts further with Cs_2_CO_3_ to provide insoluble CsCl. After this treatment, the addition of *i*-Pr_2_EtN cleanly promotes the isomerization process to provide the desired product **40**. This exemplifies that one-pot syntheses cannot be realized by the simple combination of individually optimized reaction conditions.

The next key issue was found to be the formation of an amide bond. Although the isolated carboxylic acid **41** can be coupled with the amine easily, this coupling reaction had to be performed in the presence of a phosphoric acid derivative under the current one-pot conditions, which was generated by the previous Horner–Wadsworth–Emmons reaction releasing a byproduct. In spite of the amine **42** being able to react with the phosphoric acid byproduct, we reasoned that the activated derivative of the carboxylic acid **41** would be more reactive than that derived from the phosphoric acid. Thus, the amidation reaction was performed at 0 °C to minimize the formation of phosphonamides, after which the temperature was increased to room temperature. This result indicates that it is necessary to design new reaction conditions according to the existing byproducts and current one-pot conditions, which will clearly differ from the optimized conditions using isolated starting material.

In short, the eventual synthesis developed consisted of nine reaction steps in one pot, namely: (1) an asymmetric Michael reaction of an acetaldehyde (**34** → **35**), (2) a domino Michael reaction/(3) Horner–Wadsworth–Emmons reaction (**35** → **37**), (4) a retro-aldol/(3) Horner–Wadsworth–Emmons elimination (**38** → **37**), (5) a retro-Michael reaction (**39** → **37**), (6) an isomerization (**37** → **40**), (7) the conversion of a *tert*-butyl ester to a carboxylic acid (**40** → **41**), (8) amide bond formation (**41** → **43**), and (9) a final nitro to amine group reduction (**43** → **44**).

### 8.4 Three-pot synthesis of prostaglandin E_1_ (PGE_1_) methyl ester^37^

**Scheme 5:**
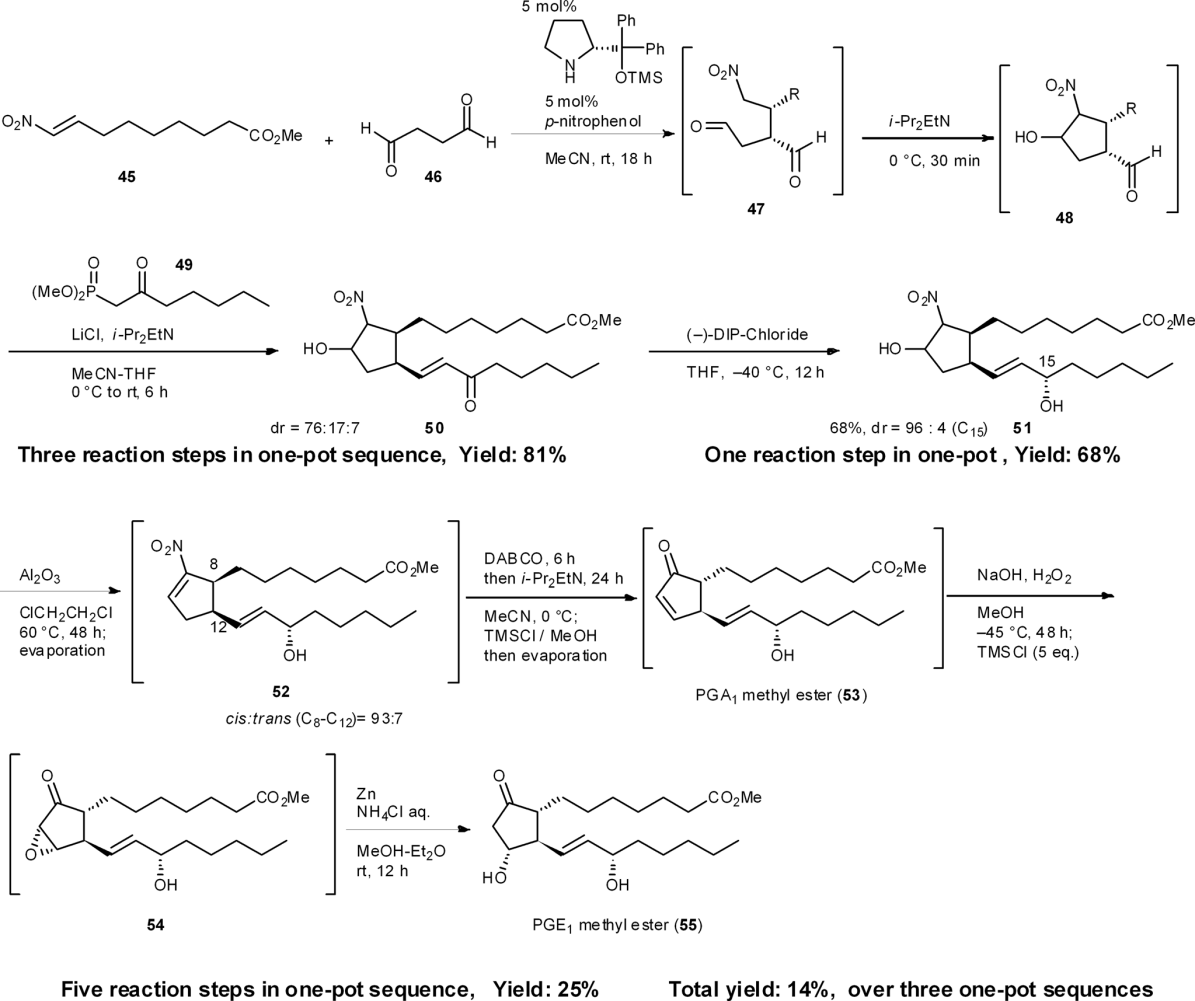
Three-pot synthesis of prostaglandin E_1_ methyl ester.

The prostaglandins are known to act as local hormones, controlling a multitude of important physiological properties in only trace amounts, and some of their derivatives are important medicines.^38^ There are several syntheses of the prostaglandins^39^ including Corey's landmark synthesis.^40^ We have accomplished the enantioselective total syntheses of PGA_1_ and PGE_1_ methyl esters in 25% and 14% total yield, respectively, in three pots, which includes three isolations and three chromatographic purifications (Scheme 5). The first one-pot reaction sequence starts with a key asymmetric formal [3 + 2] cycloaddition reaction, resulting from the domino Michael reaction of succinaldehyde **46** and nitroalkene **45**, with a successive intramolecular Henry reaction, to afford the cyclopentanecarbaldehyde **48**.^41^ A sequential Horner–Wadsworth–Emmons reaction then provides **50**, which possesses all the carbons necessary for a PGE_1_ structure. As **48** readily isomerizes, it is essential to carry out the Horner–Wadsworth–Emmons reaction in the same reactor to obtain excellent diastereoselectivity and high yields. After the diastereoselective reduction of ketone **50**, the following five reaction steps were developed to occur in a single reactor, namely: (1) a dehydration step (**51** → **52**) followed by a novel base-mediated conversion of a nitroalkene into an α,β-unsaturated ketone (**52** → **53**), comprising (2) the α,β- to β,γ-isomerization of the unsaturated nitro compound, and (3) an oxidative Nef reaction using molecular oxygen,^42^ as well as (4) an epoxidation step (**53** → **54**) and (5) the reductive opening of the epoxide (**54** → **55**). An *in situ* quenching method was also employed in this one-pot synthesis. Namely, after the base-promoted conversion of nitroalkene **52** into the α,β-unsaturated ketone **53**, the reaction mixture was neutralized by the addition of TMSCl. After the epoxidation of α,β-unsaturated ketone **53** using NaOH and H_2_O_2_, a second addition of TMSCl also neutralized the reaction mixture. Such modifications are necessary when performing several reactions in the same reactor. As the intermediate **53** was found to be unstable, *via* the facile isomerization of its double bond, this one-pot procedure, without workup, increased the yield.

### 8.5 Baclofen

**Scheme 6:**
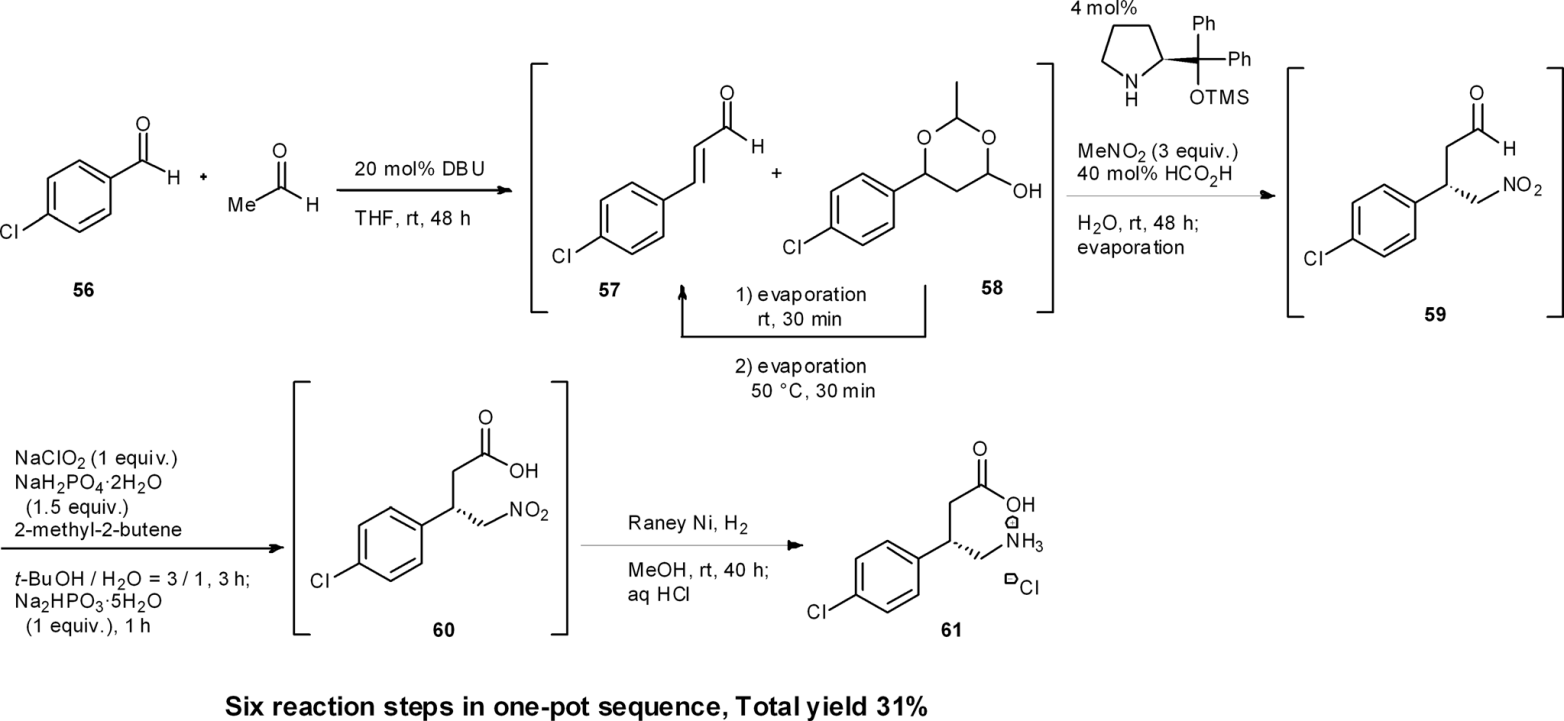
One-pot synthesis of (*S*)-baclofen.

Baclofen is a potent GABA_B_ receptor agonist used for the treatment of spinal cord injury-induced spasms.^43^ Recently we accomplished the targeted synthesis of (*S*)-baclofen from commercially available materials over four reaction steps in a one-pot set of operations (Scheme 6).^44^ The success of our one-pot synthesis was to develop an efficient synthesis of the α,β-unsaturated aldehyde that also gave conditions suitable for subsequent reactions to proceed in the same reactor. Although there are many methods for the preparation of α,β-unsaturated aldehydes from the corresponding aldehyde, there was no known method that would circumvent aqueous workups, as well as byproducts, side-products or remaining reagents deleterious to the next reactions. In particular, the next reaction was to be an asymmetric catalytic reaction, and even a small amount of side-product was expected to decrease the enantioselectivity. We soon realized that the aldol condensation of acetaldehyde would be an ideal first method because the byproduct would only be water. To this end, we developed a DBU catalyzed aldol condensation between *p*-chlorobenzaldehyde **56** and acetaldehyde. This provided the desired α,β-unsaturated aldehyde **57** (with the side-product acetal **58**). Acetal **58** could then be converted into the desired aldehyde **57** by retro-acetalization and dehydration at 50 °C. If acetaldehyde was present during the conversion of **58** to **57**, then an undesired aldol reaction of **57** with acetaldehyde would proceed. Thus, excess acetaldehyde was removed under reduced pressure and, after the conversion of **58** to **57**, the additional acetaldehyde, which was further generated *via* retro-acetalization, was also removed under reduced pressure at 50 °C. By adopting this newly developed procedure, the desired α,β-unsaturated aldehyde **57** was obtained in good yield (Section 6.5).

Another synthetic challenge was the subsequent asymmetric Michael reaction of nitromethane catalyzed by diphenylprolinol silyl ether,^45^ which had to be conducted in the presence of DBU. To quench DBU as the stronger base, the asymmetric reaction was eventually found to proceed, without compromising enantioselectivity, by adding formic acid. Subsequently, oxidation (**59** → **60**) and reduction (**60** → **61**) were carried out in the same pot. Thus, by the dual development of a new aldol condensation reaction sequence and suitable modification of the reaction conditions, a one-pot synthesis of (*R*)-baclofen was realized. This synthesis consists of six reaction steps in a single reactor, namely: (1) an aldol addition reaction, (2) a dehydration step (**56** → **57**) and (3) a retro-acetal reaction; followed by (2) dehydration (**58** → **57**), (4) asymmetric Michael reaction of nitromethane (**57** → **59**), (5) oxidation (**59** → **60**) and (6) reduction (**60** → **61**).

### 8.6 Horsfiline and coerulescine

**Scheme 7:**
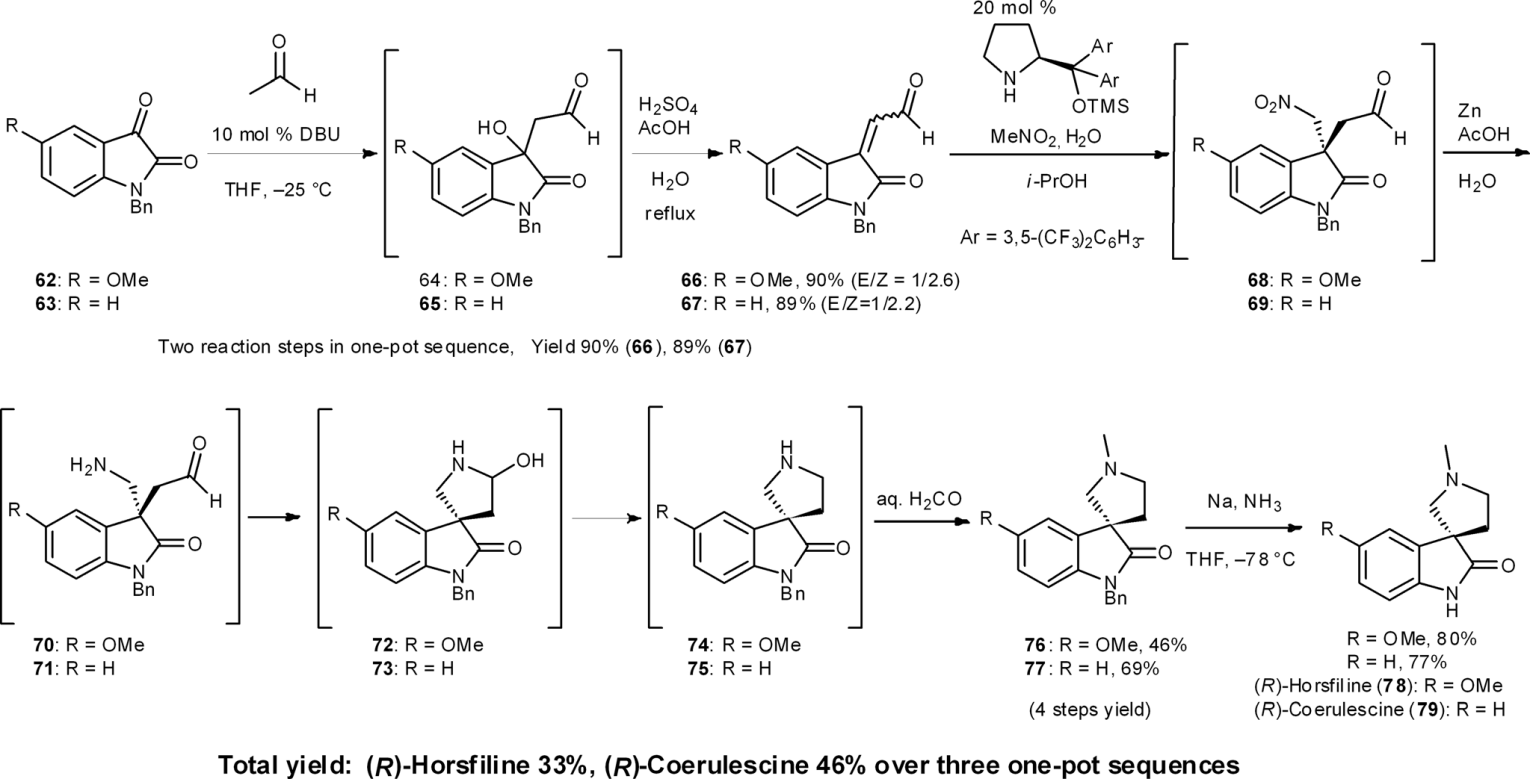
Three-pot synthesis of (*R*)-horsfiline and (*R*)-coerulescine.

(–)-Horsfiline (**78**) and (–)-coerulescine (**79**) are spirooxyindole alkaloids that have been isolated from *Horsfieldia superba* in 1991 by Bodo's group^46^ and from *Pharalis coerulescens* in 1998 by Colegate's group,^47^ respectively. In our three-pot synthesis of these alkaloids, an organocatalyzed asymmetric reaction was selected as the key step (Scheme 7).^48^ As a one-pot prelude to this key step, the first reaction is a DBU-catalyzed aldol addition with acetaldehyde and then the condensation of an isatin derivative **62**/**63**.^44^ The next reaction sequence comprises four reactions: (1) the Michael reaction of nitromethane and β,β-disubstituted aldehydes **66**/**67** catalyzed by diphenylprolinol silyl ether to generate all-carbon quaternary stereogenic centers in **68**/**69** with excellent enantioselectivity;^49^ (2) reduction of the nitro group to amines **70**/**71** in the presence of Zn and acetic acid; (3) the intramolecular reductive amination *via* aminal formation of **72**/**73** to provide the pyrrolidino-spirocycles **74**/**75**; and (4) the installation of an *N*-methyl group by sequential intermolecular reductive amination by adding formaldehyde to the reaction mixture. This sequence afforded **76** and **77** in 46% and 69% yield over four reactions in a single reactor from aldehydes **66** and **67**, respectively. In one pot, Zn acted as a reducing reagent in three different ways: (1) reduction of a nitro group to an amine (**68** → **70**, **69** → **71**), (2) intramolecular reductive amination (**70** → **74**, **71** → **75**), and (3) intermolecular reductive amination (**74** → **76**, **75** → **77**) (Section 6.6).

### 8.7 One-pot synthesis of chiral pyrans and piperidines

One-pot reaction sequences to produce high-valued intermediates efficiently are well suited for process chemistry. Moreover, one-pot syntheses are highly applicable for medicinal chemistry, because they can generate a diversity of compound types in a short period of time.

**Scheme 8:**
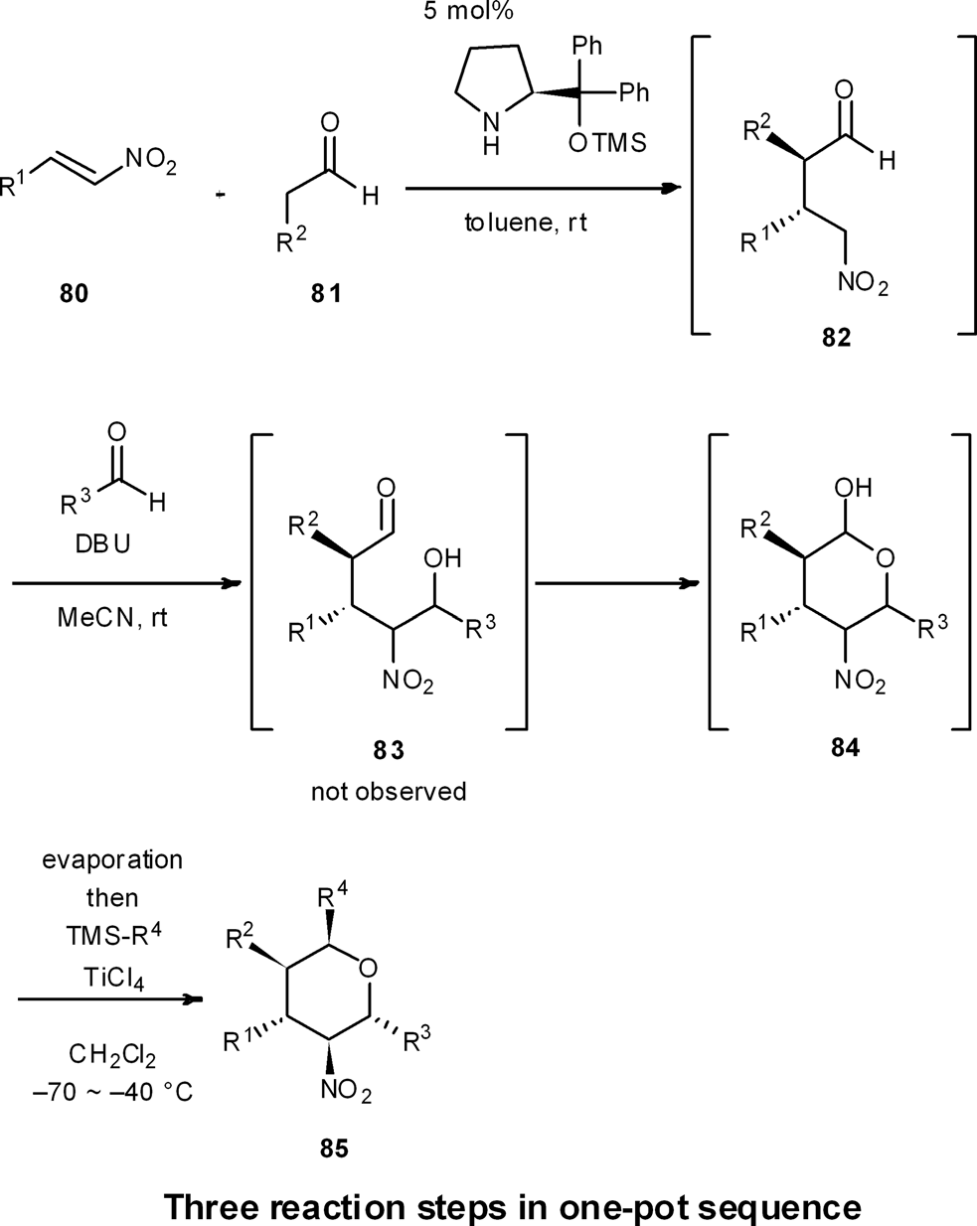
One-pot synthesis of diversely substituted tetrahydropyrans.

For instance, we have developed a four-component coupling reaction between two different aldehydes, a nitroalkene, and a silylated nucleophile to provide substituted chiral tetrahydropyrans in one pot over three reaction steps, with one final purification step (Scheme 8).^50^ The reaction starts with (1) a diphenylprolinol silyl ether mediated asymmetric Michael reaction of an aldehyde and a nitroalkene (**80** and **81** → **82**),^7^ followed by (2) a domino Henry reaction/intramolecular acetalization (**82** → **84**), and (3) a Lewis acid-catalyzed nucleophilic addition reaction (**84** → **85**). This reaction sequence proceeds with excellent diastereoselectivity and enantioselectivity, and substituents R^1^, R^2^, R^3^ and R^4^ can be readily diversified with the use of different aldehydes, nitroalkenes and nucleophiles. Here, work-up and purification is only conducted one time at the end, allowing this one-pot approach to generate a plethora of substituted chiral tetrahydropyrans in a streamlined and timely manner.

**Scheme 9:**
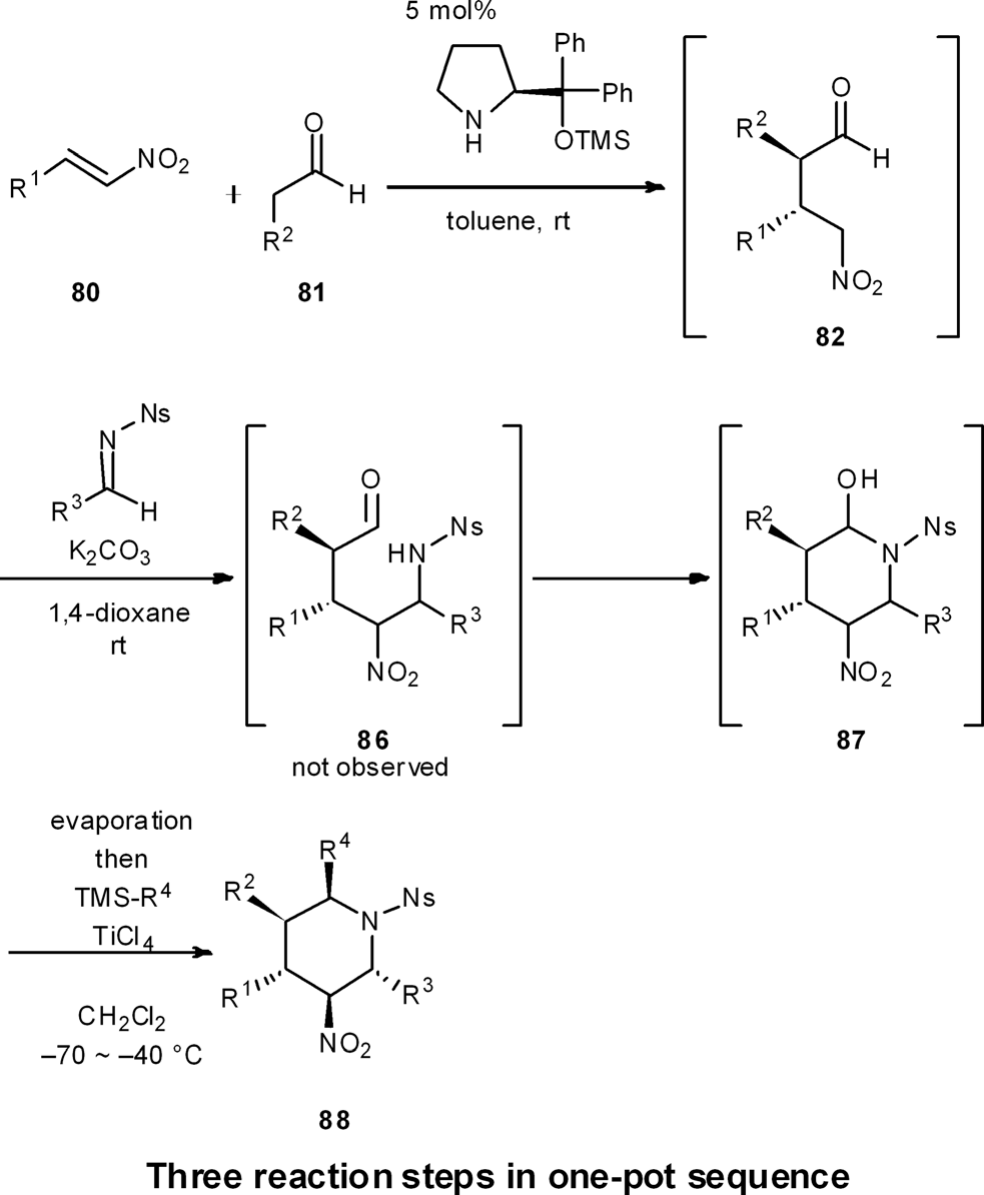
One-pot synthesis of highly substituted piperidines.

This strategy was extended to a one-pot synthesis of chiral substituted piperidines (Scheme 9).^51^ An efficient, asymmetric, four-component, one-pot synthesis of highly substituted piperidines with excellent diastereo- and enantioselectivity was thus established through the diphenylprolinol silyl ether-mediated Michael reaction of aldehyde **81** and nitroalkene **80**, followed by a domino aza-Henry/hemi-aminalization reaction sequence, and a final Lewis-acid mediated allylation or cyanation reaction. In this way, all carbons of the piperidine ring can be substituted with different groups, and the five contiguous stereocenters are completely controlled in both a relative and absolute sense. As R^1^, R^2^, and R^3^ are readily changed by adopting different starting materials, this is a concise method to generate a diverse range of chiral substituted piperidines in a relatively rapid fashion.

These two examples thus demonstrate the rapid synthesis of highly substituted tetrahydropyran and piperidine frameworks, which hold promise as chemical libraries of pharmaceutically important chiral building blocks, and further illustrate the power of one-pot syntheses in the fields of medicinal chemistry and, potentially, process chemistry.

## 9. Conclusion

The one-pot synthesis of target molecules is not new. Under an impetus to determine the most efficient strategy to synthesize a molecule, one-pot approaches have been widely used, even in the first reports of multistep organic synthesis, to carry out several reactions in the same reactor. Such pot economy encompasses the concepts not only of domino reactions, in which all the reagents are mixed together from the beginning, but also multistep reactions in which the reagents are added and changed successively, as well as *in situ* work-up procedures or quenching events that are performed in order to modify the one-pot conditions for the next reaction. These are one-pot syntheses in as far as the reactions proceed in the same reactor, but it is more than that. Several characteristics and limitations of one-pot syntheses have been described herein, mostly from the accomplishments of my own group’s studies. As demonstrated, the one-pot synthesis of a target molecule is not merely a linear combination of each optimized reaction. Rather, it requires logical changes in the reaction conditions to moderate reactivity, minimize byproducts, circumvent or reverse side-reactions, and importantly allow for the tactical selection of reagents that can play multiple roles in reactions downstream in the synthesis. A one-pot synthesis is thus not only a useful methodology to adopt for the production of organic molecules, but also a promising green approach to contemporary synthesis. The future of “pot economy” in the synthetic design and provision of complex molecules looks bright.
